# Competitive-Level Differences in Trunk and Foot Kinematics of Underwater Undulatory Swimming

**DOI:** 10.3390/ijerph19073998

**Published:** 2022-03-28

**Authors:** Takahiro Tanaka, Satoru Hashizume, Takahiko Sato, Tadao Isaka

**Affiliations:** 1 Graduate School of Sport and Health Science, Ritsumeikan University, Kusatsu 525-8577, Japan; 2 Faculty of Sport and Health Science, Ritsumeikan University, Kusatsu 525-8577, Japan; s-hashi@fc.ritsumei.ac.jp (S.H.); t-satou@fc.ritsumei.ac.jp (T.S.); isaka@se.ritsumei.ac.jp (T.I.); 3 Faculty of Rehabilitation, Biwako Professional University of Rehabilitation, Higashiomi 527-0145, Japan

**Keywords:** swimming performance, trunk, foot, kinematics, undulatory swimming

## Abstract

The foot and trunk kinematics could be associated with horizontal velocity during underwater undulatory swimming (UUS). This study aimed to compare the foot and trunk kinematic parameters during UUS between faster and slower swimmers. The three-dimensional coordinates of the markers were collected during 15 m UUS for 13 swimmers. Participants were divided into two groups based on their horizontal UUS velocity. The range of motion of the lower waist was greater for the faster swimmers than for the slower swimmers; however, no group differences were found for the foot orientation angle. Both the maximum flexion and extension angular velocities of the lower waist and maximum extension angular velocity of the chest were greater for faster swimmers than for slower swimmers. The toe vertical velocity during upward and downward kicks and horizontal displacement per kick were greater for the faster swimmers than for the slower swimmers, whereas no group difference was found for kick frequency. The increase in the long horizontal displacement per kick could be explained by the increase in vertical velocity of the great toes due to the increased trunk angular velocity. These results indicate that faster swimmers performed the UUS with greater trunk angular velocity.

## 1. Introduction

Competitive swimmers perform a whip-like movement involving their whole body under the water surface to shorten the race time, especially in freestyle, backstroke, and butterfly stroke events. This swimming style is known as underwater undulatory swimming (UUS) and is only permitted for the first 15 m immediately after the start and at each turn as per the rules of the Fédération Internationale de Natation (FINA) [1]. Competitive swimmers perform UUS more than 0.5 m below the water surface to reduce the wave drag compared with surface swimming [2,3]. Therefore, the horizontal body velocity is generally greater during UUS than during surface swimming [4]. Furthermore, previous studies have suggested that the high horizontal body velocity of UUS during the start and turn phases was a factor for improving the race time [4,5,6,7,8]. Therefore, the UUS technique is considered to be important in improving race time.

The drag and lift forces applied to the swimmer’s body as a propulsive fluid force are mainly exerted by the foot during UUS [9,10]. Previous studies suggested that a greater maximum plantar flexion angle during a downward kick would induce an increase in the foot segment angle relative to the long axis of the pool lane in the sagittal plane (i.e., foot orientation angle), resulting in the generation of a greater propulsive fluid force during a downward kick [10,11,12]. Swimmers produce a greater horizontal UUS velocity by increasing the kick frequency and decreasing or maintaining the vertical amplitude of the toe position [10,13,14]. Furthermore, the fluid force is proportional to the square of the velocity of the body; therefore, the vertical velocity of the toes is also an important parameter for increasing the propulsive force [10,15,16]. These results indicated that the foot orientation angle, toe vertical velocity, and kick frequency would induce generation of the great propulsive fluid force during UUS. Swimmers, therefore, may induce greater horizontal body velocity by a large foot orientation angle, kick frequency, and/or the vertical velocity of the toes. 

Trunk movements are thought to affect the foot movements and the horizontal velocity of the body during UUS [11,15,17,18]. A simulation study reported that a greater horizontal body velocity was found for UUS with trunk undulatory movement than without the corresponding movement [17]. Ikeda et al. [18] showed that a lower trunk angular displacement was related to horizontal body velocity during UUS, thereby indicating that a greater trunk angular displacement induces an increase in the horizontal UUS velocity. One of the possible geometrical reasons could be that the large range of motion of the trunk induces an increase in the foot orientation angle at the starting point of the downward and upward kicks, and/or the vertical amplitude of the toe position, resulting in increasing the propulsive fluid force applied to the feet [11,12,17]. Furthermore, in the whip-like movement, the larger linear and angular velocities of the proximal segment induced an increase in the corresponding velocities of the distal segment [19,20]. This indicates that a greater trunk angular velocity may also induce an increase in the vertical velocity of the toes and the kick frequency during UUS [16,17]. Therefore, the trunk range of motion and the corresponding angular velocity are important parameters for improving the horizontal body velocity during UUS. 

Based on previous studies, faster swimmers may perform UUS with great trunk range of motion and/or corresponding angular velocity to increase the foot orientation angle, toe vertical velocity, and kick frequency. Previous studies have examined the relationships between horizontal body velocity and trunk and foot kinematic parameters [15,18]. A previous study also reported that trunk kinematics could affect thigh and lower leg movements during UUS [18]. Although foot kinematics are thought to be important factors for increasing horizontal body velocity during UUS [10,11,12,14,15,16], these studies have not examined the effect of trunk movement on foot kinematics during UUS. Therefore, it remains unknown from previous studies whether faster swimmers produced a greater horizontal body velocity by increasing trunk range of motion and/or corresponding angular velocity to improve the foot orientation angle, toe vertical velocity, and/or kick frequency during UUS. This suggests that there is a lack of information on how to improve trunk movements to improve foot kinematics, which is related to greater horizontal body velocity during UUS. Understanding trunk movement strategies for improving the foot movement of faster swimmers could provide important insights regarding technical training for swimmers and coaches to increase their horizontal body velocity during UUS. The purpose of this study, therefore, was to compare the foot kinematic parameters, trunk joint range of motion, and corresponding angular velocity during UUS between faster and slower swimmers. We hypothesized that (1) the trunk range of motion and the foot orientation angle is greater for faster swimmers than slower swimmers, and (2) trunk angular velocities, toe vertical velocity, and kick frequency are also greater for faster swimmers than slower swimmers.

## 2. Materials and Methods

### 2.1. Participants

Thirteen male swimmers (age; 20.6 ± 2.40 years old, body height; 1.71 ± 0.05 m, body mass; 67.2 ± 6.21 kg.) participated in this study. The participants belonged to a collegiate swimming team or a local swimming club. The FINA points of the participants’ personal best of the long course used to evaluate the swimmer’s competitive level was 626.2 ± 81.2. The experimental protocol was approved by the local ethics committee and was conducted in accordance with the guidelines set out in the Declaration of Helsinki [21]. Written informed consent was obtained from each participant prior to the experiment.

### 2.2. Swimming Trials

**Figure 1:**
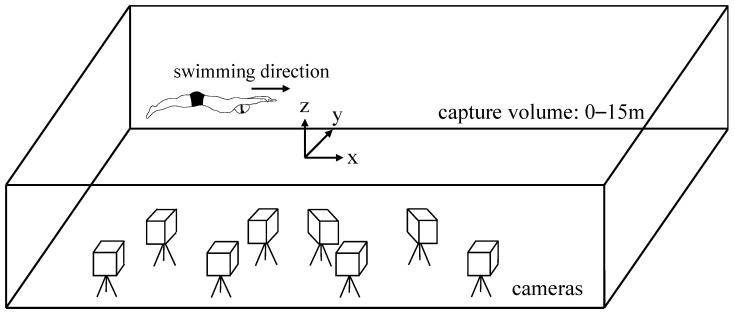
The experimental setup for the data collection.

Participants performed the 15 m UUS three times with maximum effort by using the wall-push start at an indoor pool (7 lanes × 25 m, depth; 1.35 m, water temperature; 30 °C) (Figure 1). The participants were asked to maintain their body depth (>0.75 m) from the water’s surface during the experimental trials [22,23]. Prior to the experimental trials, participants were allowed to warm up to moderate intensity for approximately 10 min. The participants rested for at least 2 min between the experimental trials to reduce the effect of fatigue.

### 2.3. Experimental Setting and Data Collection 

**Figure 2:**
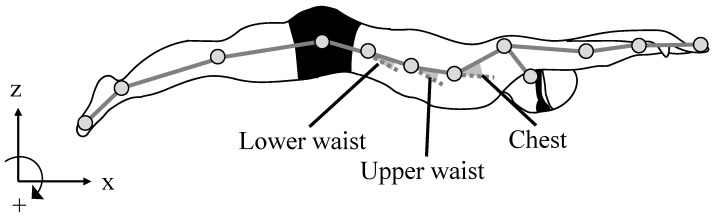
The whole-body segments model.

Twelve retro-reflective markers (16 mm) were attached to the skin over the bony configurations of the right side of the body as follows: epiphysis of the fifth metatarsal, lateral malleolus, lateral epicondyle of the femur, greater trochanter, iliac horn, the lower end of the tenth rib, xiphoid, tragus, acromion, lateral epicondyle of the humerus, styloid process, and tips of the third finger [15,17,24] (Figure 2). The three-dimensional (3D) coordinates of the markers were collected using an underwater motion capture system with eight cameras (Qualysis, Sweden) at a sampling rate of 100 Hz (Figure 1). The capture volume was set as a lane from the wall to 15 m from the wall. The error in the dynamic calibration of this system, computed as the standard deviation of the known length of the calibration wand was 1.1 ± 0.2 mm. Three successful trials were recorded for each participant. 

### 2.4. Data Analysis

The 3D coordinates were filtered using a second-order Butterworth low-pass filter with a cut-off frequency of 6 Hz [14,16]. During the UUS, the head was behind the upper arm; therefore, the 3D coordinates of the head marker were estimated using the corresponding data of the tragus and acromion [24]. The current study assumed the UUS as the symmetrical movement on the sagittal plane, which was defined by the vertical and long axes of the lane [16,24] (Figure 2). One kick cycle was defined from the instance of the highest toe marker to the instance of the next highest toe marker [14,15,24]. In the present study, kinematic analysis was conducted for the three kick cycles after the foot passed 7.5 m from the wall [25,26]. The horizontal body velocity was computed from the position of the whole-body center of mass, estimated using the body segment inertial properties of Japanese athletes [27]. The trial in which the mean horizontal velocity of the body during the three kick cycles was fastest among the three trials was chosen for additional data analysis for each participant. 

The kick frequency was determined as the reciprocal of the duration of each kick cycle. The horizontal displacement per kick was determined from the horizontal body velocity and kick frequency for each kick cycle. The vertical amplitude of the toe position was determined as the difference between the highest and lowest positions of the epiphysis of the fifth metatarsal. Thereafter, the corresponding relative value was obtained by dividing by the swimmers’ body height [14]. In each of the upward and downward kick phases, the maximum toe vertical velocity was determined using the 3D coordinates of the epiphysis of the fifth metatarsal. The foot orientation angle was calculated as the angle between the long axis of the pool lane and the line passing through the epiphysis of the fifth metatarsal and the lateral malleolus at the instant when the positions of the epiphysis of the fifth metatarsal were the highest and lowest during each kick phase. 

**Figure 3:**
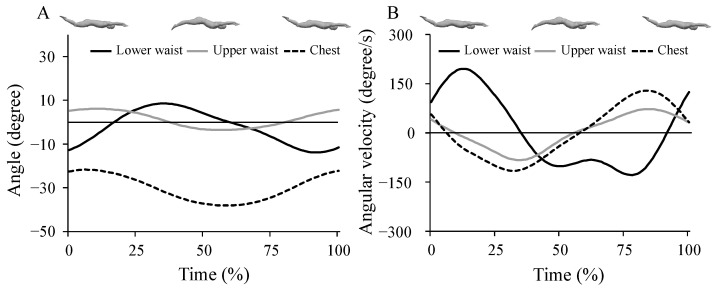
The typical time–history data of angles (**A**) and angular velocities (**B**) of the lower waist, upper waist, and chest. The solid black line, solid gray line, and the broken black line represent the lower waist, upper waist, and chest, respectively. The positive and negative values represent the flexion and extension directions, respectively. 0%, 50%, and 100% represent the starting point of the downward kick, starting point of the upward kick, and finishing point of the upward kick, respectively.

The lower waist, upper waist, and chest joint angles were defined as the relative angles between the proximal and distal segments projected onto the sagittal plane (Figure 2). The angular velocities of each joint were then determined using the joint angle data. The time–history data of the trunk joint angles and corresponding angular velocities are presented in Figure 3. The maximum extension angles of the upper waist and chest were observed during the upward kick, whereas the corresponding extension angular velocity was observed during the downward kick. The maximum flexion angle and angular velocity of the upper waist and chest were observed during an upward kick. The maximum flexion angle and angular velocity of the lower waist were observed during the downward kick, whereas the corresponding extension angle and angular velocity were observed during the upward kick. The maximum flexion and extension angles and corresponding angular velocities of each joint were determined for each kick cycle. Furthermore, the difference between the maximum flexion and extension angles in each kick cycle was determined to represent the range of motion of each joint. All kinematic variables were analyzed using MATLAB (2019a, MathWorks).

### 2.5. Statistical Analysis

Data are presented as mean ± standard deviation. The mean value of three kick cycles for each parameter was used as the representative value for each participant. The thirteen participants were divided into seven faster and six slower swimmers using the horizontal body velocity during the three kick cycles (Table 1). Participants who produced a horizontal UUS velocity equal to or greater than the median thereof for all swimmers were defined as faster swimmers. The normality of each data point was confirmed using the Shapiro–Wilk test. Normal distribution was confirmed for the body height, weight, FINA points, and all kinematic parameters; therefore, an unpaired t-test was used to compare the data between the faster and slower swimmers. Normal distribution was not confirmed for the age of the participants; therefore, the Mann–Whitney U test was conducted. A 95% confidence interval (95% CI) of the difference was determined for all outcome measures. The effect size was calculated as described by Hoplins et al. [28] (effect size: 0–0.2, 0.2–0.6, 0.6–1.2, 1.2–2.0, 2.0–4.0 for trivial, small, moderate, large, and very large, respectively) [28,29]. Statistical analyses were performed using the IBM SPSS Statistics software (ver. 26.0, IBM Corp.). Statistical significance was set at *p* < 0.05. 

## 3. Results

No significant group differences with a small effect size were found for foot orientation angle, kick frequency, vertical amplitude of toe position, and relative amplitude of toe position, whereas the horizontal displacement per kick was greater for the faster swimmers than for the slower swimmers with a large effect size (Table 2). The maximum toe vertical velocities during the upward and downward kicks were significantly greater for the faster swimmers than for the slower swimmers, with a large effect size.

The maximum extension and flexion angles of each joint did not differ between the two groups, with a trivial to moderate effect size (Table 3). For the lower waist joint, both the maximum flexion and extension angular velocities were greater for the faster swimmers than for the slower swimmers, with a large effect size (Table 4). In contrast, the angular velocities of the upper waist joint did not differ between the two groups, with a small to moderate size effect. The maximum extension angular velocity of the chest joint was significantly greater for the faster swimmers than for the slower swimmers with a large effect size, whereas the corresponding flexion angular velocity was not different between the two groups with a moderate effect size. The range of motion of the lower waist joint was significantly greater for the faster swimmers than for the slower swimmers with a large effect size, whereas no differences were found in the range of motion of the upper waist and chest joint between the two groups with a moderate effect size (Table 3).

## 4. Discussion

This study compared the differences in foot kinematic parameters, trunk joint range of motion, and corresponding angular velocity during UUS between faster and slower swimmers. The current results showed that the range of motion of the lower waist joint was greater in the faster swimmers than in the slower swimmers; however, the foot orientation angle was not different between the two groups. These results do not support our first hypothesis. The toe vertical velocity and the angular velocities of the lower waist and chest joint were significantly greater for the faster swimmers than for the slower swimmers; however, no difference was found in kick frequency between the two groups, indicating that our second hypothesis was partly supported. 

The faster swimmers showed a greater range of motion of the lower waist joint than slower swimmers during UUS. Although no statistical differences were found for the corresponding maximum flexion and extension angles between the two groups, the combination of the mean value differences in maximum flexion (8.4° greater for faster swimmers than for slower swimmers) and extension (1.2° greater for slower swimmers than for faster swimmers) angles could explain the difference in the mean value of range of motion (7.2° greater for faster swimmers than for slow swimmers). The maximum flexion angle of the lower waist joint was 8.4° greater for the faster swimmers than for the slower swimmers, whereas the corresponding extension angle was slightly greater by 1.2° for the slower swimmers than for the faster ones. These results indicate that the difference in the maximum flexion angle of the lower waist joint between the groups is thought to be the major factor explaining the difference in the corresponding range of motion. Geometrically, the greater flexion and extension angles of the lower waist induced an increase in the foot orientation angle, resulting in the production of greater horizontal body velocity due to an increase in the propulsive fluid force applied to the feet [12,17]. Nevertheless, the current results showed no differences in the foot orientation angle between the two groups. A previous study reported that a greater lower waist angle would induce a decrease in the thigh segment angle relative to the long axis of the pool lane in the sagittal plane during UUS [18]. These results suggest that the differences in the influence of the flexion and extension angles of the lower waist joint on the foot orientation angle between the two groups may be counterbalanced by the differences in the influence of the thigh segment movements. These results also suggest that faster swimmers did not perform UUS with a greater trunk joint range of motion to increase the foot orientation angle. 

The current results revealed that the extension and flexion angular velocities of the lower waist joint and extension angular velocity of the chest joint were significantly greater for faster swimmers than for slower swimmers. Furthermore, the toe vertical velocities during the upward and downward kicks were also greater for faster swimmers than for slower swimmers. Previous studies have reported that swimmers move their feet quickly by using whip-like movements during UUS [13,15,16,17]. The peak linear and angular velocities of the trunk induce an increase in the corresponding foot velocities through a whip-like movement [19,20]. The body height is a potential factor affecting foot kinematic parameters. However, it must be noted that the difference in body height was not significant across the groups. These results suggest that the greater toe vertical velocity during the upward kick for the faster swimmers was solely attributable to the greater extension angular velocity of the lower waist joint, whereas the greater toe vertical velocity during the downward kick was attributable to the greater flexion angular velocities of the lower waist joint and extension angular velocity of the chest joint (Figure 3). Due to the fluid mechanics, toe vertical velocity is one of the key parameters determining the fluid force applied to the feet [10]. Moreover, the greater toe vertical velocity potentially induces an increase in kick frequency; however, no differences were found between the two groups. Although no group difference was found for vertical amplitude of the toe position, the faster swimmers performed the UUS with a 0.03 m greater for vertical amplitude of toe position than for slower swimmers (Table 2). Geometrically, the greater vertical amplitude of the toe position induced an increase in the duration of one kick cycle. Therefore, faster swimmers performed the UUS with greater toe vertical velocity and longer duration of each kick cycle, resulting in the statistically insignificant difference in kick frequency between the two groups. In contrast, faster swimmers showed a longer horizontal displacement per kick than slower swimmers. Therefore, faster swimmers may produce horizontal body velocity by generating a large propulsion fluid force per kick. These results suggest that faster swimmers performed UUS with greater trunk joint angular velocity to increase toe vertical velocity. 

Certain limitations must be acknowledged when interpreting these results. This study assumed that the trunk and lower extremities mainly moved in the sagittal plane during UUS [15,17,24]. Based on this assumption, a two-dimensional analysis was conducted in this study; therefore, the determined trunk angles may be contaminated by the projection error. No studies have examined the projection error on the trunk angles during UUS; however, this error was shown as 3.7–6.5 degrees, as determined during other physical activities [30,31,32]. This error was smaller than the difference in the trunk angle determined in this study (Table 3). Furthermore, the reflective markers attached to the swimmers’ body induced an increase in the water braking force; therefore, the UUS movement would be different between those with and without reflective markers [33]. A previous study reported that these differences were found to be 0.06 m/s for horizontal body velocity, 0.01 Hz for stroke rate, and 0.05 m for stroke length during front crawl with 25 reflective markers attached when compared to swimmers without markers [33]. These errors were also smaller than the differences in kinematic parameters in this study (Table 2). Finally, this study had a small sample size (*n* = 13). However, a higher effect was found for outcome measures, with significant differences between the two groups. Apart from this, the data with no significant differences had moderate to negligible effect size. Moreover, the 95% confidence interval differences did not cross over zero for the data, indicating significant differences between the two groups. These limitations may have had a small effect on the main findings of this study. 

## 5. Conclusions

The main findings of this study were as follows: (1) faster swimmers showed a greater range of motion of the lower waist joint during UUS than slower swimmers; (2) the angular velocities of the lower waist and chest joints were significantly greater for the faster swimmers than for the slower swimmers, and (3) the toe vertical velocity and horizontal displacement per kick were also greater for faster swimmers than for slower swimmers, whereas no group difference was found in kick frequency. These results suggest that faster swimmers produce a greater horizontal body velocity during UUS by increasing the horizontal displacement per kick rather than increasing the kick frequency. The greater horizontal displacement per kick was obtained from the increased vertical velocity of the toes due to an increase in the trunk angular velocity. 

## Figures and Tables

**Table 1:** The basic data of the participants.

	Faster Group (*n* = 7)	Slower Group (*n* = 6)	95% CI of Differences	*p* Value	Effect Size
Age (years)	19.9 ± 0.7	21.5 ± 3.4	−4.0–2.0	0.344	0.641
Body height (m)	1.70 ± 0.1	1.70 ± 0.0	−0.07–0.05	0.677	0.220
Body mass (kg)	66.2 ± 7.7	68.3 ± 4.2	−9.91–5.72	0.567	0.303
FINA points	669.3 ± 69.5 *	578.7 ± 58.6	11.28–170.0	0.029	1.288

Abbreviations: m: meter, kg: kilogram, CI: confidence interval, FINA: Fédération Internationale de Natation; * significant level, *p* < 0.05.

**Table 2:** The horizontal body velocity, kick frequency, horizontal displacement per kick, vertical amplitude of toes position, maximum toe vertical velocity, and foot orientation angle at the highest and lowest toe position.

	Faster Group	Slower Group	95% CI of Differences	*p* Value	Effect Size
Horizontal body velocity (m/s)	1.57 ± 0.15 *	1.31 ± 0.09	0.15–0.45	0.001	2.279
Kick frequency (Hz)	2.32 ± 0.40	2.22 ± 0.29	−0.34–0.52	0.644	0.244
Horizontal displacement per kick (m)	0.69 ± 0.08 *	0.58 ± 0.07	0.02–0.20	0.025	1.328
Vertical amplitude of toes position (m)	0.49 ± 0.05	0.46 ± 0.07	−0.04–0.12	0.568	0.435
Relative vertical amplitude of toes position (%)	29.0 ± 3.0	27.1 ± 4.4	−0.03–0.06	0.373	0.473
Maximum toes vertical velocity (m/s)					
Upward kick	3.16 ± 0.18 *	2.80 ± 0.33	0.01–0.71	0.047	1.271
Downward kick	−3.89 ± 0.10 *	−3.61 ± 0.25	−0.55–−0.01	0.043	1.364
Feet orientation angle (degree)					
at highest toe position	17.3 ± 5.6	16.7 ± 2.2	−4.7–5.9	0.809	0.121
at lowest toe position	−41.7 ± 6.4	−39.8 ± 7.3	−10.4–6.4	0.611	0.268

Abbreviations: m/s: meter/second, m: meter, CI: confidence interval; * Significant level at *p* < 0.05.

**Table 3:** The maximum extension and flexion angle and range of motion of the lower waist, upper waist, and chest.

	Faster Group	Slower Group	95% CI of Differences	*p* Value	Effect Size
Lower waist					
Maximum extension (degree)	−13.8 ± 14.2	−15.0 ± 8.8	−13.6–15.9	0.867	0.088
Maximum flexion (degree)	13.1 ± 12.7	4.7 ± 11.5	−6.6–23.3	0.243	0.631
Range of motion (degree)	26.9 ± 4.75 *	19.7 ± 3.0	2.3–12.2	0.008	1.647
Upper waist					
Maximum extension (degree)	−4.0 ± 4.4	−4.0 ± 8.3	−7.9–7.9	1.000	0.000
Maximum flexion (degree)	8.0 ± 5.2	5.9 ± 8.7	−6.4–10.7	0.597	0.278
Range of motion (degree)	12.0 ± 2.79	9.85 ± 3.3	−1.6–5.8	0.237	0.639
Chest					
Maximum extension (degree)	−36.7 ± 10.2	−40.5 ± 9.6	−8.3–16.0	0.504	0.357
Maximum flexion (degree)	−17.5 ± 12.5	−25.5 ± 11.3	−6.7–22.7	0.255	0.615
Range of motion (degree)	19.2 ± 4.17	15.0 ± 3.6	−0.6–9.0	0.081	0.985

Abbreviations: CI: confidence interval; * Significant level at *p* < 0.05.

**Table 4:** The maximum extension and flexion angular velocity of the lower waist, upper waist, and chest.

	Faster Group	Slower Group	95% CI of Differences	*p* Value	Effect Size
Lower waist					
Maximum extension (degree/s)	−177.8 ± 40.9 *	−130.5 ± 28.1	−91.0–−3.7	0.036	1.225
Maximum flexion (degree/s)	217.3 ± 36.5 *	180.0 ± 13.5	2.5–72.2	0.038	1.213
Upper waist					
Maximum extension (degree/s)	−100.0 ± 21.9	−81.5 ± 23.1	−46.0–8.9	0.166	0.760
Maximum flexion (degree/s)	104.7 ± 33.7	85.6 ± 36.3	−23.6–56.7	0.383	0.465
Chest					
Maximum extension (degree/s)	−141.5 ± 28.8 *	−104.2 ± 18.6	−67.5–−7.1	0.020	1.394
Maximum flexion (degree/s)	154.3 ± 38.6	115.8 ± 30.6	−4.6–81.5	0.075	1.008

Abbreviations: CI: confidence interval; * Significant level at *p* < 0.05.

## Data Availability

Not applicable.

## References

[1] Fédération Internationale de Natation FINA Swimming Rules. https://www.fina.org/swimming/rules.

[2] Blitvich J.D., Mcelroy G.K., Blanksby B.A., Clother P.J., Peason C.T. (2000). Dive Depth and Water Depth in Competitive Swim Starts. J. Swim. Res..

[3] Elipot M., Dietrich G., Hellard P., Houel N. (2010). High-level Swimmers’ Kinetic Efficiency during the Underwater Phase of a Grab Start. J. Appl. Biomech..

[4] Veiga S., Cala A., Frutos P.G., Navarro E. (2014). Comparison of Starts and Turns of National and Regional Level Swimmers by Individualized Distance Measurements. Sports Biomech..

[5] Arellano R., Brown P., Cappaert J., Nelson R.C. (1994). Analysis of 50-M, 100-M, and 200-M Freestyle Swimmers at the 1992 Olympic Games. J. Appl. Biomech..

[6] Houel N., Elipot M., André F., Hellard P. (2012). Influence of Angles of Attack, Frequency and Kick Amplitude on Swimmer’s Horizontal Velocity during Underwater Phase of a Grab Start. J. Appl. Biomech..

[7] Takeda T., Sakai S., Takagi H. (2020). Underwater Flutter Kicking Causes Deceleration in Start and Turn Segments of Front Crawl. Sports Biomech..

[8] Veiga S., Roig A., Gómez-Ruano M.A. (2016). Do Faster Swimmers Spend Longer Underwater than Slower Swimmers at World Championships?. Eur. J. Sport Sci..

[9] von Loebbecke A., Mittal R., Mark R., Hahn J. (2009). A Computational Method for Analysis of Underwater Dolphin Kick Hydrodynamics in Human Swimming. Sports Biomech..

[10] Cohen R.C., Cleary P.W., Mason B.R. (2012). Simulations of Dolphin Kick Swimming Using Smoothed Particle Hydrodynamics. Hum. Mov. Sci..

[11] Maglischo E.W. (2003). Swimming Fastest.

[12] Sugimoto S., Nakashima M., Ichikawa H., Miwa T., Takeda T., Nomura T. (2008). The Effects of Plantar Flexion Angle Increment on the Performance during Underwater Dolphin Kick Using Simulation Analysis. Jpn. J. Phys. Educ. Health Sport Sci..

[13] Hochstein S., Blickhan R. (2014). Body Movement Distribution with Respect to Swimmer’s Glide Position in Human Underwater Undulatory Swimming. Hum. Mov. Sci..

[14] Shimojo H., Sengoku Y., Miyoshi T., Tsubakimoto S., Takagi H. (2014). Effect of Imposing Changes in Kick Frequency on Kinematics During Undulatory Underwater Swimming at Maximal Effort in Male Swimmers. Hum. Mov. Sci..

[15] Atkison R.R., Dickey J.P., Dragunas A., Nolte V. (2014). Importance of Sagittal Kick Symmetry for Underwater Dolphin Kick Performance. Hum. Mov. Sci..

[16] Higgs A.J., Pease D.L., Sanders R.H. (2017). Relationships Between Kinematics and Undulatory Underwater Swimming Performance. J. Sports Sci..

[17] Nakashima M. (2009). Simulation Analysis of the Effect of Trunk Undulation on Swimming Performance in Underwater Dolphin Kick of Human. J. Biomech. Sci. Eng..

[18] Ikeda Y., Ichikawa H., Shimojo H., Nara R., Baba Y., Shimoyama Y. (2021). Relationship between Dolphin Kick Movement in Humans and Velocity during Undulatory Underwater Swimming. J. Sports Sci..

[19] Stodden D.F., Fleisig G.S., McLean S.P., Lyman S.L., Andrews J.R. (2001). Relationship of Pelvis and Upper Torso Kinematics to Pitched Baseball Velocity. J. Appl. Biomech..

[20] Kreighbaum E., Barthels K.M. (1996). Biomechanics a Qualitative Approach for Studying Human Movement.

[21] World Medical Association (2013). Declaration of Helsinki, Ethical Principles for Scientific Requirements and Research Protocols. Bull. World Health Organ..

[22] Lyttle A.D., Blanksby B.A., Elliott B.C., Lloyd D.G. (1998). The Effect of Depth and Velocity on Drag during the Streamlined Glide. J. Swim. Res..

[23] Novais M.L., Silva A.J., Mantha V.R., Ramos R.J., Rouboa A.I., Vilas-Boas J.P., Sérgio R.L., Daniel A.M. (2012). The Effect of Depth on Drag During the Streamlined Glide: A Three-Dimensional CFD Analysis. J. Hum. Kinet..

[24] Yamakawa K.K., Shimojo H., Takagi H., Tsubakimoto S., Sengoku Y. (2017). Effect of Increased Kick Frequency on Propelling Efficiency and Muscular Co-activation during Underwater Dolphin Kick. Hum. Mov. Sci..

[25] Arellano R., Pardillo S., Gavilán A. Underwater Undulatory Swimming: Kinematic Characteristics, Vortex Generation and Application During the Start, Turn and Swimming Strokes. Proceedings of the XXth International Symposium on Biomechanics in Sports.

[26] Connaboy C., Coleman S., Moir G., Sanders R. (2010). Measures of Reliability in the Kinematics of Maximal Undulatory Underwater Swimming. Med. Sci. Sports Exerc..

[27] Ae M., Tang H., Yokoi T. (1992). Estimation of Inertia Properties of the Body Segments in Japanese Athletes. Biomechanism.

[28] Hopkins W.G., Marshall S.W., Batterham A.M., Hanin J. (2009). Progressive Statistics for Studies in Sports Medicine and Exercise Science. Med. Sci. Sports Exerc..

[29] Cohen J. (1988). Statistical Power Analysis for the Behavioral Sciences.

[30] Brown S.J., Selbie W.S., Wallace E.S. (2013). The X-Factor: An Evaluation of Common Methods Used to Analyse Major Inter-segment Kinematics during the Golf Swing. J. Sports Sci..

[31] Smith A.C., Roberts J.R., Wallace E.S., Kong P., Forrester S.E. (2016). Comparison of Two- and Three-Dimensional Methods for Analysis of Trunk Kinematic Variables in the Golf Swing. J. Appl. Biomech..

[32] Soutas-Little R.W., Beavis G.C., Verstraete M.C., Markus T.L. (1987). Analysis of Foot Motion during Running Using a Joint Coordinate System. Med. Sci. Sports Exerc..

[33] Washino S., Mayfield D.L., Lichtwark G.A., Mankyu H., Yoshitake Y. (2019). Swimming Performance is Reduced by Reflective Markers Intended for the Analysis of Swimming Kinematics. J. Biomech..

